# Supplementary material for: Antimicrobial Effect and Probiotic Potential of Phage Resistant Lactobacillus plantarum and its Interactions with Zoonotic Bacterial Pathogens
Source: Foods. 2019 Jun 5;8(6):194. doi: 10.3390/foods8060194 (PMC6616511; doi:10.3390/foods8060194)
Supplement: Supplementary file 1 [file foods-08-00194-s001.pdf]

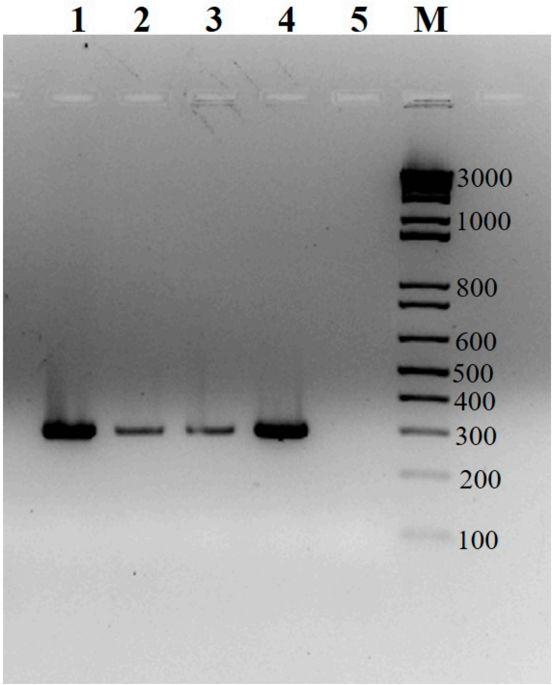

Figure S1. PCR confirmation of LP^{+PR} strain. 1% Agarose gel electrophoresis analysis of total DNA extracted from bacteriophage J1 (lane 1), LP^{+PR} strains (lanes 2-4), and wild-type LP strain (lane 5). M, 1 kb marker ladder.
